# Complete mitogenome of *Cerithidea obtusa*, the red chut-chut snail from the Cần Giờ Mangrove in Vietnam

**DOI:** 10.1080/23802359.2018.1532832

**Published:** 2018-10-26

**Authors:** Duc Hung Nguyen, Claude Lemieux, Monique Turmel, Van Duy Nguyen, Jean-Luc Mouget, Andrzej Witkowski, Réjean Tremblay, Romain Gastineau

**Affiliations:** a Faculty of Natural Sciences Pedagogy, Saigon University, Hồ Chí Minh City, Vietnam;; b Département de biochimie, de microbiologie et de bio-informatique, Institut de Biologie Intégrative et des Systèmes, Université Laval, Québec, Canada;; c Mer, Molécules, Santé (MMS), Le Mans Université, Le Mans, France;; d Faculty of Geosciences, Natural Sciences Research and Educational Center and Palaeoceanology Unit, University of Szczecin, Szczecin, Poland;; e ISMER, Université du Québec à Rimouski, Québec, Canada

**Keywords:** Cerithioidea, mangrove, Vietnam, mitogenome, Cerithidea

## Abstract

We sequenced the complete mitogenome of the red chut-chut snail *Cerithidea obtusa*, from the Cần Giờ mangrove in Vietnam. The mitogenome is 15,708 bp long. It is colinear with the mitogenomes of other members of the superfamily Cerithioidea, and the maximum-likelihood phylogeny obtained with the *cox*1, *cox*2 and *cox*3 genes of several Caenogastropoda associated all Cerithioidea together inside a strongly supported clade.

*Cerithidea obtusa* (Caenogastropoda, Cerithioidea, Potamididae) (Lamarck, 1822) is an aquatic gastropod commonly found in South-East Asia, with a broad distribution ranging from Madagascar to Australia (Reid et al. 2013, 2014). This snail generally lives in mud flats and mangroves and is famous for its peculiar behavior of climbing on tree tops during tidal inundation and letting itself fall on the mud. Known as ‘ốc len’ in Vietnam, *C. obtusa* has economic and gastronomic value. It is considered a delicatessen by inhabitants of the Cần Giờ Mangrove Biosphere Reserve, near Hồ Chí Minh City, who cook it in coconut milk.

We obtained a specimen of *C. obtusa* from a local fisherman (10°25′55″N; 106°54′530″E), extracted total DNA and sent this preparation to the Beijing Genomic Institute (Shenzhen) for DNA sequencing on the BGISEQ-500 platform. A total of 50 million paired-end reads of 100 bp were assembled using SPAdes 3.12.0 (Bankevich et al. 2012) with a k-mer value of 55. The shell of the individual used for sequencing remains in the collection of the Đại học Sài Gòn – Saigon University. The remaining flesh of the individual and the rest of the DNA extracted for sequencing are stored in the University of Szczecin (Poland) at –20 °C.

The 15,708-bp mitogenome sequence of *C. obtusa* (GenBank: MH682098) is the first complete mitogenome reported for a member of the Potamididae family. It contains 37 genes that encode two rRNAs, 22 tRNAs and 13 proteins. Coding sequences are located on both DNA strands and protein-coding genes are found in the following order: *cox*1, *cox*2, *ND4L*, *ND4*, *ND5*, *cox*3, *cob*, *ND6*, *ND1*, *ND2*, *atp*8, *atp*6, *ND3*. The *C. obtusa* mitogenome is similar in size and colinear with its homologues in the superfamily Cerithioidea (KT153076, KF736848, KJ696780, KU221394, KU878411 and LC006055); however, it differs in gene order relative to other mitogenomes of Caenogastropoda (Cunha et al. 2009; Rawlings et al. 2010). A maximum-likelihood tree was inferred using MEGA6 (Tamura et al. 2013) and three concatenated genes (first and second codon positions of *cox*1, *cox*2 and *cox*3) from 45 taxa of Caenogastropoda. *Cerithidea obtusa* was found to be part of a highly supported clade containing all eight taxa sampled from the Cerithioidea (Figure 1).

**Figure 1.**
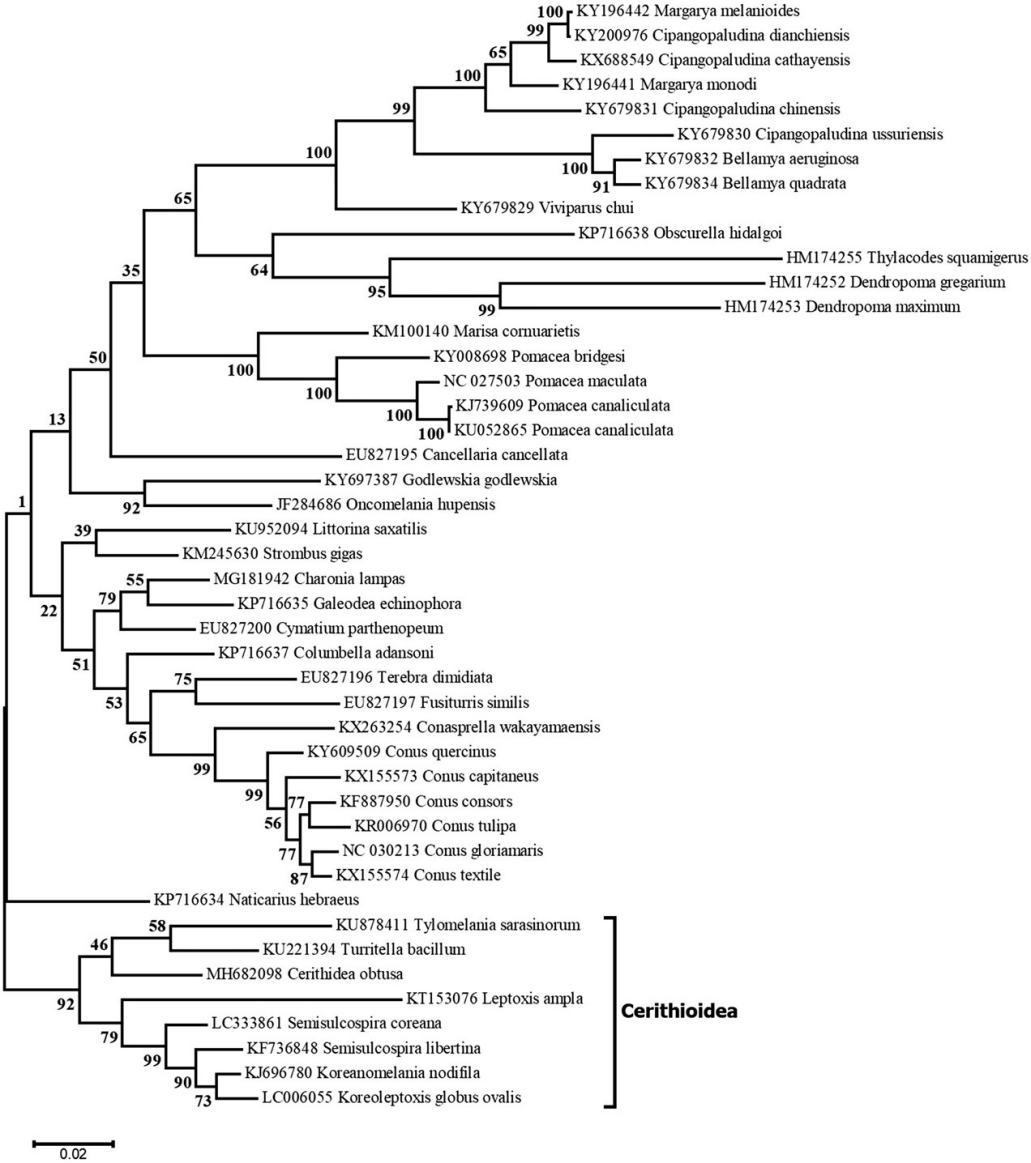
Maximum-likelihood tree of concatenated *cox1*, *cox2* and *cox3* genes of various Caenogastropoda. The tree with the highest likelihood is shown, with bootstrap values obtained after 1000 replications. The bracket indicates the position of the superfamily Cerithioidea.
